# *Croton oligandrus* Pierre & Hutch (Euphorbiaceae) Extracts and Isolated Compounds Reverse HIV-1 Latency

**DOI:** 10.2147/JEP.S472234

**Published:** 2024-11-19

**Authors:** Chantal Emade Nkwelle, Smith B Babiaka, Clovis S Metuge, Kimberly Liang, Unique Stephens, Seraphine Nkie Esemu, David S Zuzga, Kristy Shuda McGuire, Luis J Montaner, Roland N Ndip, Ian Tietjen, Fidele Ntie-Kang

**Affiliations:** 1 Department of Microbiology and Parasitology, Faculty of Science, University of Buea, Buea, Cameroon; 2 Center for Drug Discovery, Faculty of Science, University of Buea, Buea, Cameroon; 3 Department of Chemistry, Faculty of Science, University of Buea, Buea, Cameroon; 4 Department of Microbial Bioactive Compound, University of Tübingen, Tübingen, Germany; 5 Drug Discovery, The Wistar Institute of Anatomy and Biology, Philadelphia, PA, USA; 6 Institute of Pharmacy, Martin-Luther University Halle-Wittenberg, Halle, Germany

**Keywords:** antiviral, compounds, extracts, HIV-1, latency reversal, screening

## Abstract

**Background:**

*Croton oligandrus* Pierre & Hutch is a tropical tree that grows in West and Central Africa, used in ethnomedicine to treat cancer, diabetes, headaches, convulsions, urinary diseases, and inflammatory diseases. As other *Croton* species have been observed to possess chemical compounds that target HIV latency-reversal, we hypothesized that this species may have similar properties.

**Aim of the Study:**

The identification of extracts and compounds of this species, which have HIV-1 latency-reversing activity in J-Lat T cell lines.

**Methods:**

The stem bark was obtained, air-dried, powdered, and extracted using dichloromethane. In vitro flow cytometry was used to monitor GFP expression, a marker of HIV latency reversal, following treatment of J-Lat T cells with extracts and compounds.

**Results:**

Four extracts were found to reverse HIV latency, the most active extract showing better activity (ie, latency reversal in 69.7 ± 7.1% [mean ± s.e.m.] of J-Lat 10.6 cells at 1 µg/mL) than control agents prostratin (46.2 ± 9.5% at 1.2 µg.mL) and the “Mukungulu” (*Croton megalobotrys*) extract (34.9 ± 24.2% at 1 µg/mL). Extracts reversed HIV latency through mechanisms over and above protein kinase C (PKC) activation and distinct from histone deacetylase (HDAC) inhibition. The most active extract also synergized with the control HDAC inhibitor romidepsin but did not synergize with other extracts. Isolated compounds (β-Stigmasterol and lupeol) had limited but consistent latency reversal on their own.

**Conclusion:**

The plant extracts and compounds reverse HIV latency through mechanisms additional to PKC activation and/or synergize with romidepsin in vitro. Extracts and compounds from this plant may enhance the activity of current HIV latency-reversing agents being assessed in HIV cure studies.

## Introduction

According to the World Health Organization, HIV and AIDS continue to be a major global health problem, being responsible for ~630,000 deaths and 1.3 million new infections in 2023 alone.^1^ While combination antiretroviral therapy (cART) is widely implemented and has successfully reduced worldwide morbidity and mortality, cART only inhibits active HIV replication.^2^ Notably, cART fails to target resting CD4+ T cells containing latent HIV proviruses, or HIV reservoirs, which can reactivate at any time to produce infectious virus. Identifying and eradicating these latent reservoirs is necessary to achieve a long-term, cART-free HIV remission or HIV cure.

To date, the only successful cure strategy is stem cell transplantation using HIV resistant CCR5Δ32 stem cells.^3–5^ However, this approach is not practical for widespread treatment for persons living with HIV, particularly those living in low- and middle-income countries. In addition to other strategies like HIV vaccines, another experimental strategy toward achieving cART-free HIV remission or HIV cure involves the use of latency-reversing agents (LRAs) that induce HIV-1 provirus expression from latent reservoirs.^6^ This HIV reactivation, coupled with immunotherapy support, could render infected cells “visible” to the host immune system for elimination,^7^,^8^ whereas co-administration of cART would prevent further seeding of viral reservoirs.^9–11^ This approach, frequently termed “shock-and-kill” or “kick-and-kill”, could theoretically eliminate an individual’s viral reservoir and/or reduce the viral reservoir to a point that cART-free remission is achievable, provided that sufficiently effective LRAs and immune enhancers can be identified.

**Figure 1:**
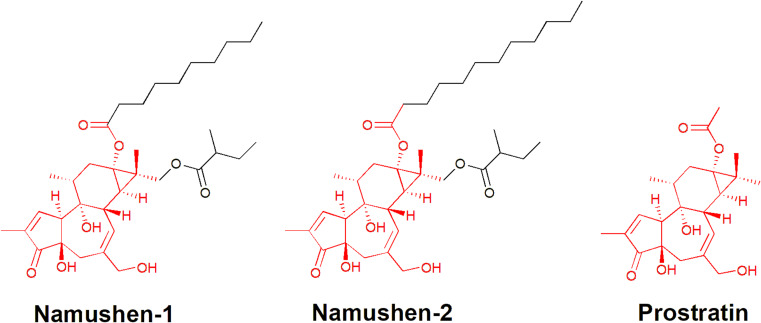
Chemical structures of components of *Croton megalobotrys* Müll Arg (Namushen-1 and Namushen-2), that show close similarity with the reference protein kinase C activator Prostratin. The substructure of the protein kinase C activator/modulator (prostratin) has been highlighted in the chemical structures of the phorbol esters from Mukungulu.

Natural products obtained from plants are diverse chemical compounds and include both HIV-1 inhibitors and novel LRAs.^12^ *Croton* is a large genus of the family Euphorbiaceae, which comprises over 1300 species of trees, shrubs, and herbs, distributed across tropical and subtropical regions.^13^ *Croton* species are widely reported for other bioactivities including anti-inflammatory, antiulcer, hypolipidemic, hypoglycemic, nephroprotective, myorelaxant, and anticonvulsant properties.^14^ While many *Croton* species have been used traditionally for centuries, they have more recently gained attention as sources of bioactive compounds against HIV. Notably, several *Croton* species have been identified to have both anti-HIV replication and/or HIV latency-reversing properties.^15–23^ In *Croton* species, HIV latency reversal is driven in large part by phorbol esters. For example, the natural product prostratin activates viral gene transcription in latently infected cells by stimulating protein kinase C (PKC) signaling, which results in the nuclear translocation of NF-κB and initiation of HIV-1 proviral transcription.^24^,^25^ Furthermore, crude extracts from *Croton megalobotrys* Müll Arg. (“Mukungulu”), used traditionally for HIV/AIDS management in northern Botswana, can induce HIV expression in vitro and contain phorbol esters called namushens 1 and 2, with structural similarity to prostratin (Figure 1).^23^

To the best of our knowledge, no study has assessed the sister species *C. oligandrus*, which is a tropical tree belonging to the Crotonidaea subfamily that grows up to 10 meters tall and is restricted to West and Central African forests.^26^ Pieces of this plant are used in local ethno-medicine for treatment of cancers, diabetes, headache, convulsion, urinary diseases and inflammatory diseases.^18^,^27^ The stem bark of this plant is also used in ethno-medicine by the Pygmy peoples in Gabon to treat anemia and for colic, stomach disorders, pneumonia and splenomegaly.^28^ However, subsequent research on the species collected in Cameroon showed that the primary class of metabolites was clerodane-type diterpenes.^14^ The stem bark and leaves collected in the Central region of Cameroon, Nkol-nkoumou, were found to contain clerodane, labdane and trachylobane diterpenes; crotonoligaketone, crotonadiol, imbricatadiol, crotonzambefuran B, 7-acetoxy trachiloban-18-oic acid, and 3-*O*-acetyl aleuritic acid.^29^,^30^ It was also reported that the stem bark collected in Bafut, North West region of Cameroon, contained the clerodane diterpenoids crotoliganfuran and 12-*epi*-crotocorylifuran, as well as scopoletin, geddic acid, sitosterol, vanillic acid, and stigmastane-3,6-dione.^27^ However, chemical compounds that may contribute to HIV latency reversal from this plant have not been determined.

In this study, we report the HIV-1 latency reversing potential of *C. oligandrus* Pierre & Hutch extracts, their mechanism of action, synergistic activity of the extracts with control LRAs, and the isolation and latency reversing activity of single compounds from bioactive extracts.

## Materials and Methods

### Laboratory Reagents

Prostratin and GÖ-6983 were purchased from Sigma-Aldrich (St. Louis, MO, USA). Romidepsin was purchased from Selleck Chemicals (Houston, TX, USA). Resazurin and Trypan blue were purchased from Mediatech, Inc. (Manassas, VA 20109 USA). Jurkat cells (Clone E6-1) were obtained from the American Type Culture Collection (ATCC; Manassas, VA, USA). J-Lat Full Length cells (clones 10.6 and 6.3), a Jurkat-derived T cell line model of HIV latency containing a full-length, envelope-defective, integrated HIV-1 genome that expresses a green fluorescent protein (GFP) reporter upon activation, were obtained from the NIH AIDS Reagent Program, Division of AIDS, NIAID, NIH (contributed by Dr. Eric Verdin).^31^ J-Lat 10.6 and J-Lat 6.3 cells differ in the genomic integration site of the HIV-1 genome; J-Lat 10.6 cells have a weak barrier to viral reactivation while J-Lat 6.3 cells are more resistant to viral reactivation by LRAs. Betulin, β-stigmasterol, and lupeol were purchased from Sigma-Aldrich (St. Louis, MO, USA).

Jurkat and J-Lat cells were maintained in R10+ medium consisting of RPMI-1640 (Life Technologies, Grand Island, NY, USA) supplemented with 10% fetal bovine serum (GeminiBio, Sacramento, CA, USA), 1% penicillin/streptomycin (Mediatech, Manassas, VA, USA), and 1% L-Glutamine (Mediatech). Cultured cells were incubated at 37 °C and 5% CO_2_. Extracts and compounds were dissolved in 100% DMSO and stored at −20 °C for further analysis.

### Plant Material

The stem bark of *C. oligandrus* Pierre & Hutch (Euphorbiaceae) was harvested in Nkol-Nkoumou, Centre Region of Cameroon, in October 2019. The plant was identified by Mr. Victor Nana, of the National Herbarium, Yaoundé, Cameroon, where a voucher specimen (No. 6687/SRF/Cam) has been deposited. The bark of *Croton megalobotrys* (“Mukungulu”) was obtained and prepared as described previously.^22^

### Extraction and Isolation

The air-dried powdered (2.2 kg) stem bark of *C. oligandrus* was extracted with dichloromethane three times for 72 h at room temperature. Filtration and concentration of the crude extract on a rotary evaporator led to 24.4 g of a dark brown crude extract. The crude extract was subjected to silica gel normal phase open column chromatography and eluted with a gradient system of EtOAc in hexane (5:95 to 100 EtOAc) followed by 100% dichloromethane to afford 75 fractions, which were combined into nine pooled fractions (A−I) based on thin-layer chromatography (TLC) profiles. Column chromatography was carried out with glass columns using Merck 60 (60–200 μm) silica gel as stationary phase. Size-exclusion chromatography was performed with Sephadex LH-20 (Sigma Aldrich). Analytical TLC was performed on Merck F254 aluminum sheets pre-coated with silica gel. Zones on these plates were visualized under UVGL-58 lamps at 254/365 nm. The ^1^H and ^13^C NMR spectra were recorded in CDCl_3_ at 400 and 100 MHz respectively, with TMS as the internal reference. The chemical shifts (δ) of carbon and proton are in parts per million (ppm), with TMS as reference (Tables S1-S3).

### HIV Latency Reversal Assays

HIV latency reversal assays were performed as described previously.^23^ Briefly, J-Lat 10.6 or 6.3 cells were resuspended in fresh R10+ medium to a concentration of 1 million cells/mL. 2×10^5^ cells (ie cells in a volume of 200 µL) were seeded into each well of a 96-well sterile, flat-bottom plate (Corning, USA) with extracts or compounds at concentrations presented in Figures and/or 0.1% DMSO vehicle control in duplicate. For each experiment, positive controls included established LRAs (prostratin – PKC activator and romidepsin – HCAC inhibitor) and/or Mukungulu extract. Cells were incubated at 37 °C and 5% CO_2_ for 24 hours. Cell cultures were then assessed for GFP expression by flow cytometry using a FACSCelesta Flow Cytometer (BD Bioscience, Franklin Lakes, NJ, USA). Flow cytometry data were analyzed using FlowJo v. 10.10.0 software (FlowJo LLC, Ashland, OR, USA). Gating of live cells and percent HIV latency for each well were obtained as described previously.^23^,^31^ For dose–response curves and synergism studies, results are reported as the mean ± s.e.m. from at least 3 independent experiments. Studies using GÖ-6983 reflect the mean ± SD from 2 independent experiments.

### Cell Viability Assays

Jurkat cells were resuspended in fresh R10+ to a concentration of 10^6^ cells/mL, and 2×10^5^ cells were seeded in 96-well plates with extracts or compounds at concentrations presented in Figures and/or 0.1% DMSO vehicle control in duplicate. Mukungulu extract was used as a control. Cells were incubated at 37 °C and 5% CO_2_ for 24 hours. Cells were then treated with 0.2 mg/mL resazurin and incubated for an additional 4 hours, and resorufin production was measured using an Infinity M200 multimode plate reader (Tecan Life Sciences; Morrisville, NC, USA). The average background fluorescence of media-only controls was subtracted from the fluorescence obtained from each well. The fluorescence from each well was then normalized to the average fluorescence of cells treated with 0.1% DMSO vehicle control in duplicate such that, for a given test agent, 100% denotes viability equal to viability of untreated cells (ie treated only with 0.1% DMSO vehicle control), and 0% denotes complete cell death. Results are reported as the mean ± s.e.m. from at least 3 independent experiments.

### Cytokine Detection Assays

Five million Jurkat cells were diluted in 1 mL of R10+ and incubated with extracts for 24 hours. Cell culture supernatants were then tested for the presence of cytokines (IL-2 and IL-6) using the ab270883–Human IL-2 SimpleStep ELISA® Kit and the ab178013 human IL-6 SimpleStep ELISA® Kit (Abcam, Cambridge, MA, USA) as directed by the manufacturer. Prostratin and Mukungulu were used as positive controls. Results were normalized to cells treated with 0.1% DMSO vehicle control and reported as the mean ± s.e.m. from 4 independent experiments.

### Histone Deacetylase Assays

Histone deacetylase (HDAC) activity was examined using the HDAC-Glo I/II Assay Kit (Promega; Madison, WI, USA) as per manufacturer’s instructions. Briefly, HDAC reactions were performed in white 384-well plates with 20 μL final volume/well. Extracts were diluted to desired concentrations in the provided buffer and added to wells. Jurkat cells were then resuspended in Phenol-Red- and FBS-free RPMI 1640 and seeded into wells at 3,000 cells/well. Cells treated with 0.1% DMSO served as a baseline HDAC activity control, while wells containing only media were included to control for signal background. Romidepsin was used as a positive control. Following incubation at 37 °C for 90 min, 20 μL of HDAC-Glo I/II Reagent plus 1% Triton-X100 (prepared as per manufacturer’s instructions) was added to each well. Plates were mixed for 30s, then incubated at room temperature for 30 min. Luminescence was detected by an Infinity M200 multimode plate reader (Tecan Life Sciences; Morrisville, NC, USA). Resulting data were then normalized to the no-inhibitor control following background subtraction. Results are reported as the mean ± SD from 2 independent experiments.

## Results and Discussion

### *Croton oligandrus* Extracts Reverse HIV Latency in vitro

A total of 6 extracts (UB/CA8 – 13) were obtained from *C. oligandrus* as described in Materials and Methods. To assess the HIV latency-reversing potential of these extracts, we first made use of J-Lat 10.6 cells, which are derived from the Jurkat T cell line but contain a latent, non-infectious HIV clone with a frameshift mutation in *env* (envelope glycoprotein). This latent provirus also carries an integrated, but transcriptionally latent HIV proviruses with a green fluorescent protein (GFP) reporter instead of *nef* accessory gene.^31^ As a result, HIV latency reversal can be monitored using GFP expression by flow cytometry.

**Figure 2:**
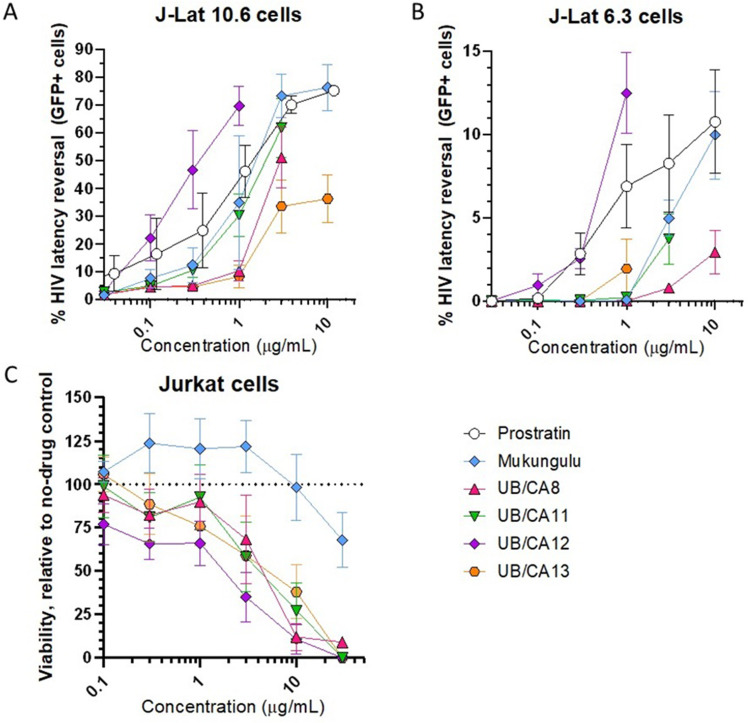
*C. oligandrus* Pierre & Hutch extracts reverse HIV latency in vitro. (**A** and **B**), Dose–response curves of control LRAs prostratin and Mukungulu extract in addition to *C. oligandrus* extracts in J-Lat 10.6 (**A**) and J-Lat 6.3 (**B**) cells. Values indicate percent GFP-positive cells for each condition. (**C**), Dose-response profiles of control LRAs and *C. oligandrus* extracts on Jurkat cell viability. Values indicate viability relative to cells treated with 0.1% DMSO vehicle control.

Consistent with published data,^23^,^32–34^ the control LRA prostratin, which reverses HIV latency through PKC activation, exhibited dose-dependent expression of GFP across multiple concentrations in J-Lat 10.6 cells after 24 hours of incubation, and where 10 µM (11.6 µg/mL) induced GFP expression in 75.2 ± 1.8% of cells (Figure 2A). Similarly, a crude extract of “Mukungulu”, isolated from *Croton megalobotrys*, a related species found in Botswana and used locally and traditionally for HIV/AIDS management,^23^ also showed a dose-dependent expression of GFP in J-Lat cells where, for example, 10 µg/mL Mukungulu reversed HIV latency in 76.3 ± 8.2% of cells (Figure 2A). Assuming a maximum induction of 75% GFP-positive cells in this model, we calculated half-maximal effective concentrations (EC_50_s) of 1.1 µg/mL for both prostratin and Mukungulu.

Using this assay, we found that 4 of 6 extracts obtained from *Croton oligandrus* also reversed latency, in some cases with more activity than prostratin and Mukungulu (Figure 2A). For example, the most potent extract, UB/CA12, induced 69.7 ± 7.1% GFP expression at only 1 μg/mL, corresponding to an EC_50_ of 0.2 μg/mL. Extracts UB/CA8, 11, and 13 also reversed HIV latency, albeit at higher concentrations corresponding to EC_50_s of 2.9, 1.2, and 3.2 μg/mL, respectively (Figure 2A). These results demonstrate that, like other *Croton* species,^16^,^23^ extracts from *C. oligandrus* also reverse HIV latency in vitro.

We next assessed whether these extracts could reverse HIV latency in J-Lat 6.3 cells, where the provirus is located at a different genomic integration site and is less likely to undergo latency reversal.^31^ In these cells, we observed that both prostratin and Mukungulu maintained the ability to reverse HIV latency, where 10 μM and 10 μg/mL induced GFP expression in 10.8 ± 3.1% and 10.0 ± 2.6% of cells, respectively (Figure 2B). Assuming a maximum 11% GFP induction in this model, the EC_50_s for prostratin and Mukungulu were calculated as 1.2 and 4.9 μg/mL. Also as observed previously, UB/CA12 remained the most active, with 12.5 ± 2.4% latency reversal at 1 µg/mL and a calculated EC_50_ of 0.5 μg/mL (Figure 2B). We also observed weaker but consistent activity for UB/CA8 (2.9 ± 1.3% at 10 μg/mL), UB/CA11 (3.7 ± 1.5% at 3 μg/mL), and UB/CA13 (1.9 ± 1.8% at 3 μg/mL; Figure 2B). While these activities were insufficient to accurately calculate EC_50_s, they demonstrate that extracts from *C. oligandrus* promote latency reversal in vitro independent of proviral integration site.

Notably, higher concentrations of each extract (*eg* > 10 μg/mL) resulted in lower GFP expression in cell cultures (not shown), indicating the potential for cytotoxicity at these concentrations. To test this rigorously, we next treated Jurkat cells, the parental cell line of J-Lat cells, in the presence of each extract and control for 24 hours and measured cell viability by resazurin stain (Figure 2C). While no toxicity was observed with Mukungulu except at 30 µg/mL (ie, 67.9 ± 15.8% viability relative to cells treated with 0.1% DMSO vehicle control), all 4 extracts exhibited dose-dependent toxicity, with calculated half-maximal cytotoxic concentrations (CC_50_s) of 7.1, 9.9, 0.9, and 9.2 μg/mL for extracts UB/CA8, 11, 12 and 13, respectively (Figure 2C), indicating that the bioactive chemical components of *C. oligandrus* found in these extracts likely differ from those of Mukungulu.

### Extracts Reverse HIV Latency in Part Through PKC Activation

**Figure 3:**
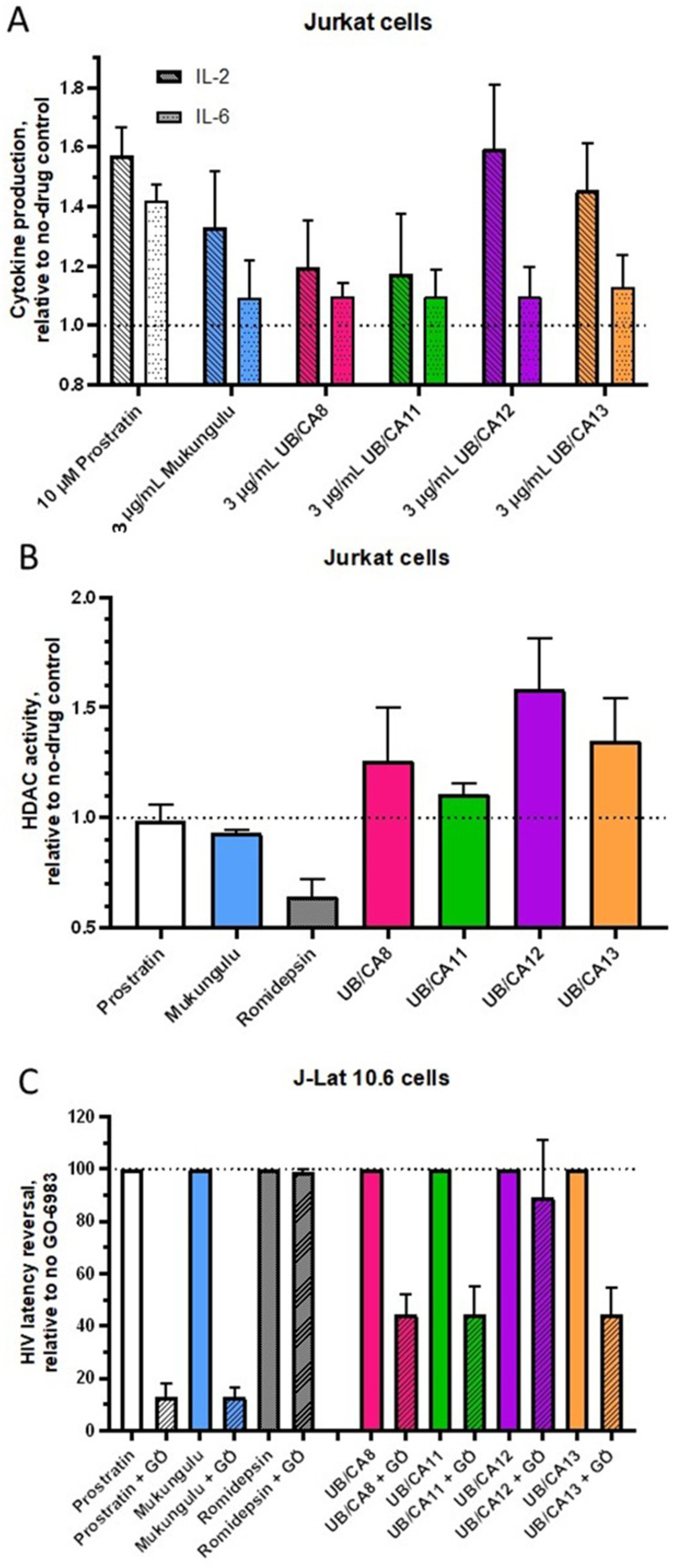
Mechanisms of *C. oligandrus* Pierre & Hutch extracts. (**A**), Effects of control LRAs and *C. oligandrus* extracts on supernatant IL-2 and IL-6 levels in Jurkat cells as measured by ELISA. Data are presented as fold-change in cytokine levels relative to untreated cells. (**B**), Effects of control LRAs and extracts on cellular HDAC activity in Jurkat cells, as measured by HDAC-Glo assay. (**C**), Effects of control LRAs and extracts in J-Lat 10.6 cells in the presence of pan-PKC inhibitor GÖ-6983.

To investigate the mechanisms of latency reversal induced by *C. oligandrus* extracts, we first assessed whether they elicited T cell activation. We therefore treated Jurkat cells with extracts or control LRAs for 24 hours and assessed culture supernatants for IL-2 and IL-6 production. In this assay, we observed that 10 µM prostratin induced a 57.2 ± 9.4% increase in IL-2 and a 41.9 ± 5.7% increase in IL-6, while 3 µg/mL Mukungulu induced 33.0 ± 18.8 and 9.5 ± 12.5% increases in these cytokines (Figure 3A). Similarly, all four tested extracts also induced IL-2 and IL-6 production, most notably by 3 µg/mL of UB/CA12 which induced a 59.5 ± 0.2% increase in IL-2 and 9.7 ± 9.7% increase in IL-6 (Figure 3A). These results indicate that, like known PKC activators prostratin and Mukungulu, these extracts also promote T cell activation in vitro.

We next asked whether these extracts may reverse HIV latency through HDAC inhibition, a common and well-established pharmacological mechanism of latency reversal.^23^ To test this, we used a previously described HDAC-glo assay to measure HDAC activity in Jurkat cells.^35^ In this assay, 0.1 µM of the control HDAC inhibitor romidepsin inhibited 35.6 ± 8.0% of HDAC activity in Jurkat cells (Figure 3B). In contrast, neither 10 µM prostratin nor 3 µg/mL Mukungulu inhibited HDAC activity (maximum 7.1 ± 1.4% inhibition), consistent with their functions as PKC activators (Figure 3B). Similarly, no inhibition was observed with any *C. oligandrus* extract (Figure 3B), indicating that these extracts are unlikely to function as HDAC inhibitors to reverse HIV latency.

To determine whether these extracts may reverse HIV latency through PKC activation, we next treated J-Lat cells in the presence of 1 µM of the pan-PKC inhibitor GÖ-6983. As expected, latency reversal induced by both 10 µM prostratin and 3 µg/mL Mukungulu were inhibited in the presence of GÖ-6983 by 87.0 ± 5.0% and 87.5 ± 4.0%, respectively (Figure 3C). Conversely, latency reversal induced by 0.1 µM romidepsin remained unaffected by co-administration of GÖ-6983 and as expected for a histone deacetylase inhibitor (1.4 ± 1.4% inhibition). Interestingly, GÖ-6983 inhibited only ~55% of latency reversal induced by 3 µg/mL of UB/CA8, 11, and 13 and 11.0 ± 22.3% of latency reversal induced by UB/CA12 (Figure 3C). These results indicate that the active components of these extracts likely contain PKC activators, but that other mechanisms of latency reversal may also be present.

### A Subset of Extracts Synergize with Romidepsin but Do Not Synergize with Each Other

**Figure 4:**
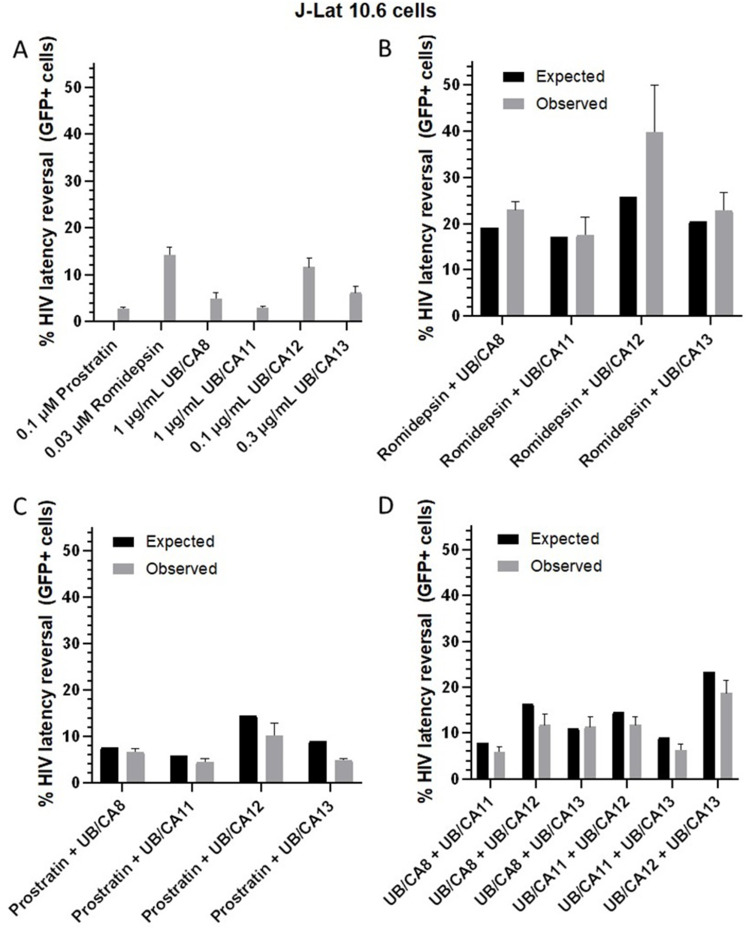
Combinatorial effects of *C. oligandrus* Pierre & Hutch extracts. (**A**), Effects of control LRAs and *C. oligandrus* extracts at sub-optimal concentrations. (**B**–**D**), Effects of extracts in combination with 0.03 µM romidepsin (**B**), 0.1 µM prostratin (**C**), and in combination with each other (**D**). For (**B**–**D**), black bars denote predicted additive activities based on results in (**A**), while gray bars denote observed activities.

Treatment of cell-line models of HIV latency with combinations of LRAs acting through different mechanisms tends to result in synergistic responses, while treatment with combinations of LRAs with similar mechanisms tends to yield only additive responses.^36–39^ To investigate whether latency reversal by *C. oligandrus* extracts also have synergistic activity, J-Lat 10.6 cells were treated with sub-maximal concentrations of UB/CA8, 11, 12, or 13 in the presence of control LRAs romidepsin (ie, HDAC inhibitor) and prostratin (ie, PKC activator; Figure 4). Using this approach, we observed that 0.1 µg/mL of UB/CA12, when combined with 0.03 µM romidepsin, resulted in greater latency reversal that what would be expected due to strictly additive effects (Figure 4B). For example, we observed that 0.1 µg/mL of UB/CA12 and 0.03 µM romidepsin on their own reversed latency in 11.6 ± 2.0% and 14.3 ± 1.6% of cells, respectively (Figure 4A), suggesting an additive activity of ~25.9% GFP expression (Figure 4B, black bar). In contrast, co-administration of these LRAs resulted in 39.8 ± 10.3% GFP expression, or ~1.5-fold more than expected by additive effects (Figure 4B, gray bar). While these results did not reach statistical significance according to the Bliss Independence model,^38^ they are consistent with UB/CA12 acting via mechanism(s) distinct from HDAC inhibitors (ie, Figure 3B). In contrast, results consistent with additive effects were observed for all extracts when combined with 0.1 µM prostratin (Figure 4C), which is also consistent with at least some of the extracts’ activities due to PKC activation (ie, Figure 3C). Finally, when extracts were tested in respective combinations, we only observed additive effects (Figure 4D), suggesting that the compounds in these extracts are likely to be functionally similar. We want to emphasize that this study includes an initial effort to isolate bioactive compounds from *C. oligandrus* extracts. The isolated compounds are likely those which are more abundant and relatively easy to isolate. We also prioritized these as they were commercially available. Clerodane diterpenoids and phorbol esters are probably also present, but they represent more complex structures and rarer species which will require more involved techniques.

### Isolation and Identification of Compounds with LRA Properties

**Figure 5:**
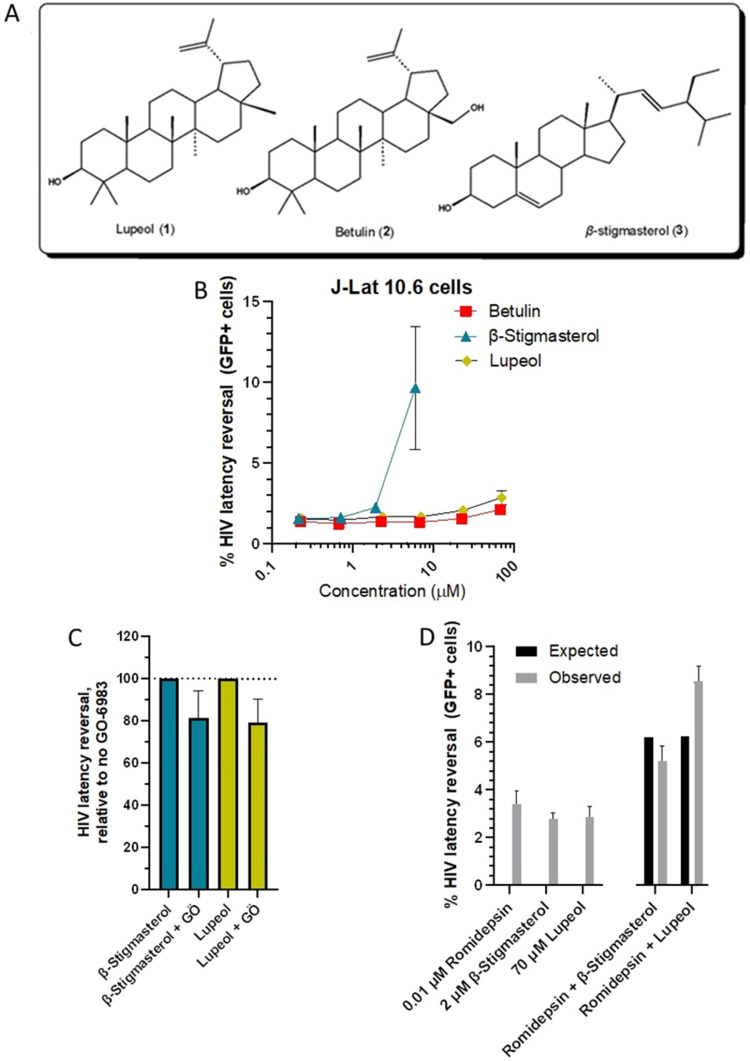
Compounds isolated from *C. oligandrus* Pierre & Hutch extracts reverse HIV latency. (**A**) Chemical structures of isolated compounds. (**B**), Dose–response curves of compounds in J-Lat 10.6 cells. (**C**), Effects of compounds in J-Lat 10.6 cells in the presence of pan-PKC inhibitor GÖ-6983. (**D**), Effects of compounds in combination with 0.01 µM romidepsin, where black bars denote predicted additive effects, and gray bars denote observed activities.

In an initial effort to identify bioactive compounds, the crude dichloromethane extract of *C. oligandrus* was subjected to silica gel column chromatography and eluted with a gradient of ethyl acetate in hexane. Repeated column chromatography through Sephadex LH-20 and TLC yielded the known phytosterols lupeol (**CO1**), betulin (**CO2**), and β-stigmasterol (**CO3**), with their masses 200.0 mg, 45.0 mg, and 23.0 mg respectively, from fraction B after further purification (Figure 5A). The compounds were obtained as white, amorphous powders and generated positive reactions for phytosterols.^40^ The structures of the **CO1**^41^ **CO2**^42^ and **CO3**^43^ were established based on NMR analyses as well as by comparison with published data (see Figures S1–S6 and Tables S1–S3).

Interestingly, when commercially available and pure versions of these compounds were assessed for latency reversal in J-Lat 10.6 cells, we saw weak but consistent activity for β-stigmasterol, where 2.5 µg/mL (6.0 µM) induced GFP expression in 9.6 ± 3.8% (Mean ± SD) of J-Lat 10.6 cells (Figure 5B). Unfortunately, higher concentrations could not be assessed due to solubility limitations. In contrast, minimal activity was observed with 30 µg/mL of betulin (67.6 µM; average 2.1 ± 0.2% GFP-positive cells) and lupeol (70.3 µM; average 2.9 ± 0.4% GFP-positive cells; Figure 5B). However, the activities of β-stigmasterol and lupeol were only minimally inhibited by co-administration with 1 µM of GÖ-6983 (18.5 ± 12.6 and 20.8 ± 11.3% inhibition, respectively; Figure 5C), indicating that these compounds are unlikely to act primarily as PKC activators. Additionally, co-administration of 70.3 µM lupeol with 0.01 µM romidepsin induced 8.6 ± 0.6% GFP expression, or a 1.4-fold increase over what would be expected by strictly additive effects (*ie*, 6.2% GFP expression, or 2.8 ± 0.4% due to 70.3 µM lupeol + 3.4 ± 0.5% due to 0.01 µM romidepsin; Figure 5D). In contrast, co-administration of 2 µM β-stigmasterol with 0.01 µM romidepsin resulted in 5.2 ± 0.6% GFP expression, which was more consistent with activity due to additive effects (Figure 5D). Taken together, these results indicate that isolated compounds have a limited but consistent ability to reverse HIV latency in vitro with mechanisms consistent with what is observed in bioactive extracts.

## Conclusion

Taken together, these results show that enriched fractions from *C. oligandrus* Pierre & Hutch, like those from other *Croton* species such as *Croton megalobotrys* (“Mukungulu”), can reverse HIV latency in vitro. However, *C. oligandrus* extracts differ from Mukungulu in that they induce more cellular toxicity and that latency reversal is less affected by PKC inhibition, suggesting the presence of distinct mechanisms of action to reverse HIV latency. We also observed that extract UB/CA12 exhibited synergistic activity when co-treated with the HDAC inhibitor romidepsin; as such, compounds isolated from this extract may be useful for enhancing the activity of current HIV LRAs like romidepsin being assessed in the clinic. Of three isolated compounds, β-stigmasterol had good activity in J-Lat 10.6 cells at 6 µM (9.6 ± 3.8% latency reversal), while betulin and lupeol had weak but detectable latency reversal above background signal (Figure 5B). Notably, these compounds were not largely affected by PKC inhibition and/or exhibited synergism with romidepsin, supporting distinct mechanisms of action from HDAC inhibitors and PKC activators. We note that other compounds including clerodane diterpenoids and/or phorbol esters are likely to be present in *C. oligandrus* extracts. Future studies should isolate and test these for latency reversing activity as they are more likely to represent the primary latency reversing activities seen here, as opposed to the compounds identified here which may function as “enhancers” of primary LRAs. Nevertheless, additional phytosterols and other compounds present in *C. oligandrus*, as well as synthetic derivatives of these compounds, should also be assessed using in vitro HIV latency as well as primary CD4+ T cell models to more closely monitor cytotoxicity, identify compounds with maximal activity, and investigate how their mechanisms of latency reversal differ from more established LRAs, which in turn would inform future in vivo studies.

## Abbreviations

cART, combination antiretroviral therapy; HDAC, histone deacetylase; HIV, human immunodeficiency virus; LRA, latency-reversing agent; PKC, protein kinase C.
